# Case Report and Review of Literature: Familial Malignant Pleural Mesothelioma in a 39 Years Old Patient With an Inconclusive ^18^F-FDG PET/CT Result

**DOI:** 10.3389/fsurg.2022.819596

**Published:** 2022-03-14

**Authors:** Mayar Idkedek, Kareem S. Tahayneh, Firas Abu-Akar, Izzeddin A. Bakri

**Affiliations:** ^1^Medical Research Club, Faculty of Medicine, Al-Quds University, Jerusalem, Palestine; ^2^Department of Cardiothoracic Surgery, Al-Makassed Charitable Society Hospital, Jerusalem, Palestine; ^3^Department of Pathology, Al-Makassed Charitable Society Hospital, Jerusalem, Palestine

**Keywords:** pleural mesothelioma, familial, BAP1 mutation, PET scan, uniportal VATS

## Abstract

Malignant pleural mesothelioma (MPM) is a rare yet aggressive neoplasm that was linked only to asbestos exposure for decades, although familial clusters were diagnosed with MPM without a known history of asbestos exposure most likely due to genetic susceptibility. Here, we describe a case of familial malignant mesothelioma in a 39 years old patient with a confirmed BAP1 mutation in addition to a known family history with the same mutation. The patient presented with progressive shortness of breath and recurrent pleural effusions and diagnosis was made through biopsies taken during uniportal Video-Assisted Thoracoscopic Surgery. After the inconclusive result of ^18^F-FDG PET/CT scan, subxiphoid uniportal Video-Assisted Thoracoscopic Surgery left pleural and laparoscopic peritoneal biopsies were obtained for staging and evaluating contralateral lung and peritoneal cavity. Finally, two important educational values should be acquired from this case: genetic predisposition and BAP1 tumor suppressor gene mutation might affect the age of presentation and overall prognosis of the disease. Also, ^18^F-FDG PET/CT scan may not be the best modality for staging and confirming the diagnosis of malignant pleural mesothelioma.

## Introduction

Malignant pleural mesothelioma (MPM) is a rare primitive type of malignancy that arises from the mesothelial and submesothelial pleural cells. It is considered an aggressive neoplasm with poor prognosis and challenging diagnostic and therapeutic measures (1).

The clinical presentation of MPM includes a wide range of constitutional symptoms; like fatigue and weight loss, or non-specific respiratory symptoms such as: dyspnea, cough, shortness of breath, and chest pain. Hence, MPM is difficult to diagnose but has been almost always linked to patients having respiratory symptoms with a known history of asbestos exposure (1).

Imaging studies may reveal pleural changes including pleural thickening, pleural effusion, calcifications, or plaques (1).

Although asbestos exposure has been believed to be the leading cause for MPM for decades (2), the incidence rate in asbestos miners is low, and this may be explained by genetic predisposition of MPM or increased sensitivity to asbestos, which need careful investigations and asbestos exposure correlation assessment (3, 4).

Additionally, familial clustering of MPM in different patterns has been recognized and reported in family members with no known asbestos exposure, which is considered the etiology of MPM (3).

Familial MPM has not been studied enough and lacks management recommendations (5). Reported literature poses the role of pathogenic germline variants in the BAP1 tumor suppressor gene, which is also responsible for BAP1 tumor predisposition syndrome (BAP1-TPDS), a syndrome characterized by several types of neoplasms, including malignant mesothelioma, uveal and keratinocytic melanoma (6).

Here, we report a case of familial malignant pleural mesothelioma (FMPM) that was diagnosed to have a BAP1 mutation and a family history of the same mutation.

The patient presented with progressive shortness of breath and huge pleural effusion and was diagnosed by a multidisciplinary team through Uniportal VATS biopsies and laparoscopy.

## Case Description

A 39 years old female patient with a positive family history of mutated BAP1 gene, and confirmed BAP1 mutation: *chr3.52406884A* >*G, c.604T* > *C, p. Trp202Arg*, was complaining of shortness of breath due to recurrent right sided pleural effusion, which required four times thoracocentesis during the last 5 years, two of which were in 2020.

The patient's history started 5 years ago when she started complaining of shortness of breath on exertion that limited her exercise tolerance to 100 meters.

The patient had no history of weight loss, fever, night sweats, chest pain, cough, sputum production, abdominal pain or distention.

On physical examination, the only finding was decreased breath sounds on the right side.

The patient had a history of similar condition in the family, the first family member: her maternal uncle died before diagnosis and he was suffering from recurrent pleural effusions and ascites while her paternal uncle died just after diagnosis with mesothelioma and was confirmed to have BAP1 mutation, which raised the suspicion of the first relative having mesothelioma due to similar presentation of recurrent effusions.

**Figure 1:**
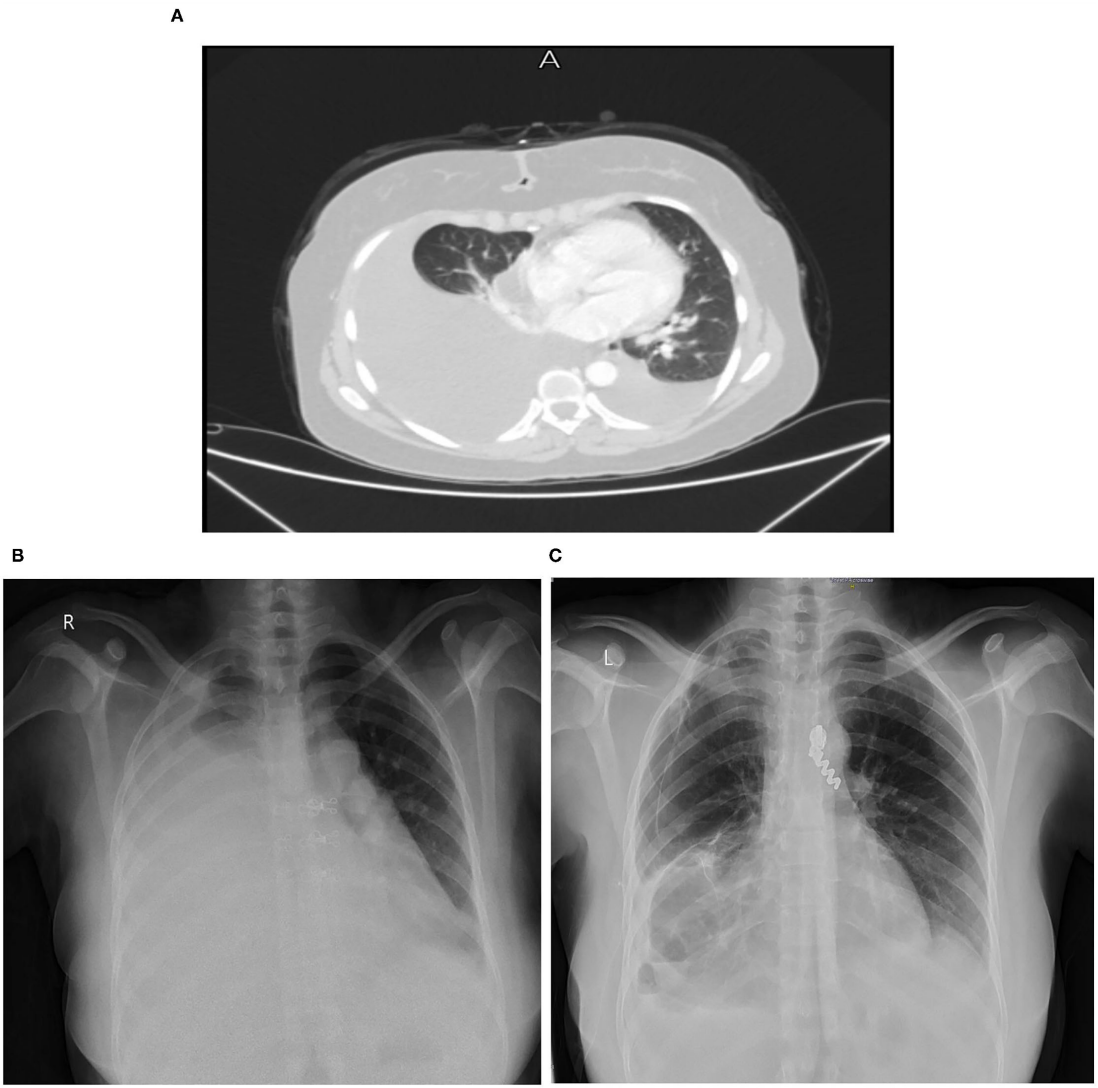
**(A)** Chest CT scan on August 17, 2020. **(B)** Chest X-ray on December 14, 2020. **(C)** Chest X-ray on April 17, 2021.

On August, 2020, contrast-enhanced computed tomography (CT) of chest revealed a huge right-sided pleural effusion causing left mediastinal shift, thickened pleura and a collapsed lung (Figure 1A).

Abdomino-pelvic CT scan showed enlarged left hepatic pole, splenomegaly, para-aortic lymph nodes and ovarian cyst.

A follow up CT scan on October, 2020 showed a large amount of partially loculated right pleural effusion with a small amount of left pleural effusion.

After multiple failed attempts of diagnosing the patient by cultures and pleural fluid cytology, the patient was referred to undergo a Uniportal VATS on October 26, 2020, aiming at evacuating right pleural effusion and taking pleural and lung biopsies for diagnosis. Laboratory evaluation showed leukocytosis (WBCs count = 19.4) and high neutrophils count = 17.6; infection was ruled out, but this may be due to the inflammatory process at the time of surgery or due to malignancy.

**Figure 2:**
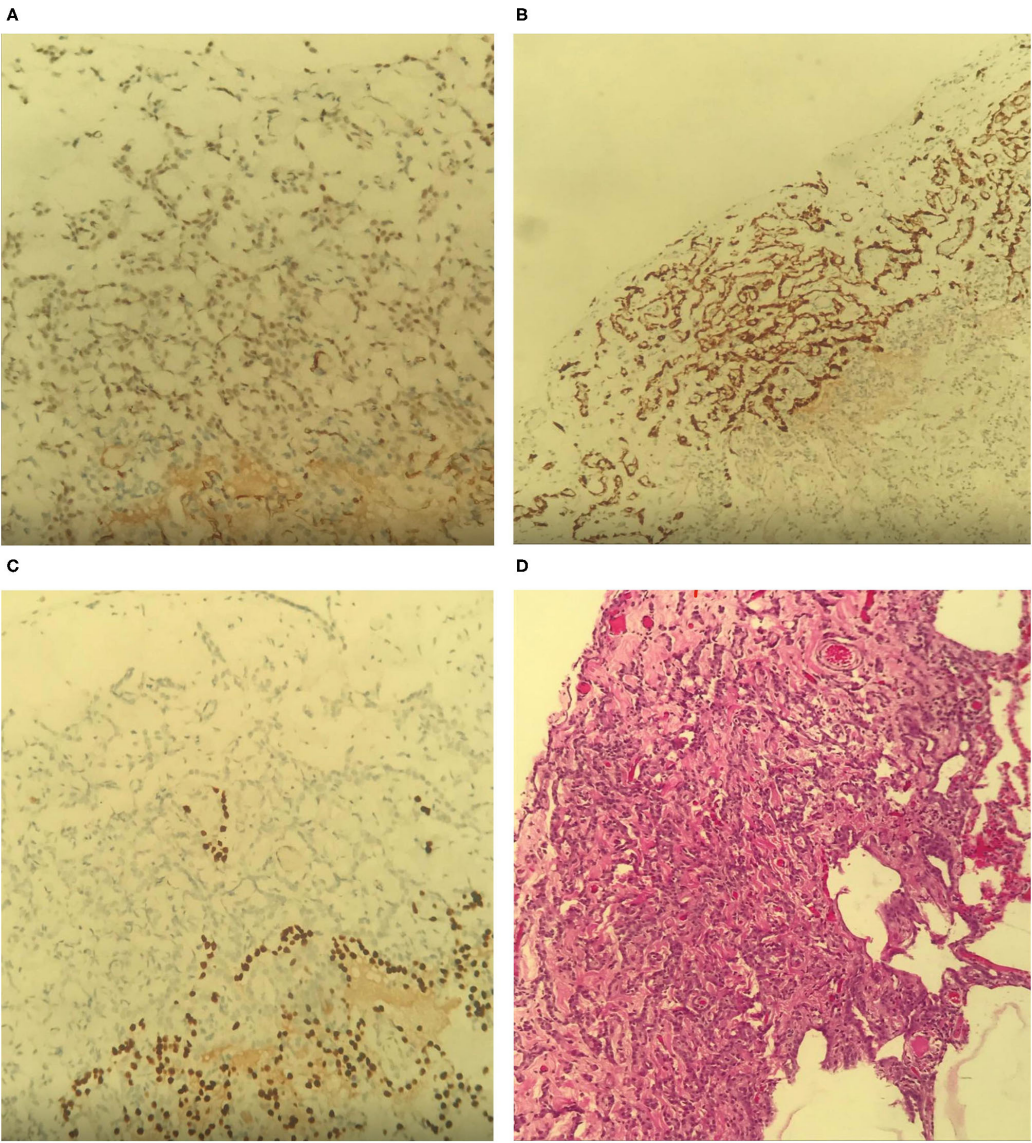
**(A)** WT1. **(B)** Calretinin. **(C)** TTF1. **(D)** H&E (10x).

According to pathology report, the submitted tissues from the right upper and lower lung lobes biopsies showed atypical mesothelial proliferation highlighted by cytokeratin 5/6, Wilms' Tumor 1 (WT1) (Figure 2A), Calretinin (Figure 2B), Pan-cytokeratin (Pan-CK) and mesothelin immunostains. However, thyroid transcription factor 1 (TTF1) was negative (Figure 2C). On hematoxylin and eosin stain (H&E), the submitted pleural tissue showed atypical mesothelial proliferation with predominance of trabecular/compressed tubular growth pattern within a sclerotic stroma (Figure 2D), and the pattern of growth was acinar with grade I nuclear features (well-differentiated).

The mitotic count is (0–1) mitosis/10 HPF (score 1), Ki67 LI: low and focal minimal invasion of the lung highlighted by pan-ck immunostain is noted.

Cytology of the right pleural fluid revealed sheets of mesothelial cells with moderate nuclear atypia in addition to pigment laden macrophages.

The overall findings of biopsies inclined toward malignant pleural mesothelioma, epithelioid subtype with low-grade features, without infiltration to underlying tissue (lung or chest wall).

**Figure 3:**
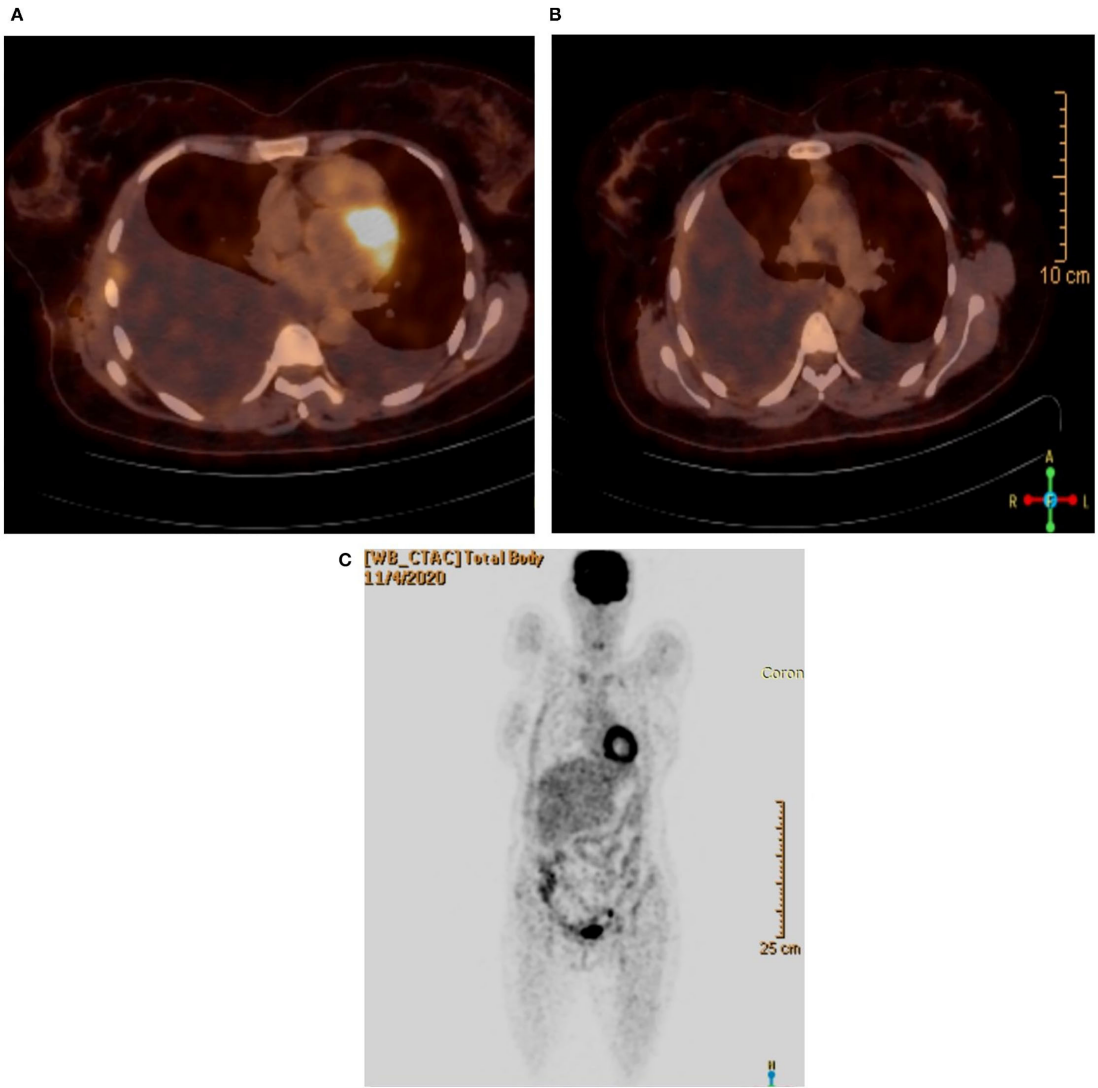
**(A)** PET/CT scan showing the lungs. **(B)** PET/CT scan without hypermetabolic activity. **(C)** Coronal view.

On November 4, 2020, ^18^F-FDG PET/CT whole body scan (Figures 3A–C) was done before taking left pleural and peritoneal biopsies in order to evaluate for pre-operative staging and possible metastasis. Unfortunately, the result was inconclusive: there was no evidence of hypermetabolic pulmonary or pleural nodules. It only showed marked right sided pleural effusion with atelectatic changes and mild left sided pleural effusion.

On December 14, 2020, a chest x-ray (Figure 1B) and CT scan were done again for follow up, which revealed a large amount of right sided pleural effusion causing left mediastinal shift and right lung collapse, with mild left pleural effusion, and a small amount of pericardial effusion, but no significant mediastinal, hilar or axillary lymphadenopathy were noted. There was no pulmonary parenchymal mass or consolidation detected or definite pleural thickening or nodularity that suggests mesothelioma. Laboratory evaluation showed high WBCs count = 17.6 and a high neutrophils = 13.9. On 15/12, CRP was 26.3 and neutrophils count fell to 7.6.

After PET/CT scan failed to give information about staging, on December, 2020, the patient was admitted again to the hospital to do a subxiphoid uniportal VATS for left pleural and laparoscopic peritoneal biopsies for staging the disease, evaluating contralateral lung and chest cavity, in addition to the peritoneal cavity.

**Figure 4:**
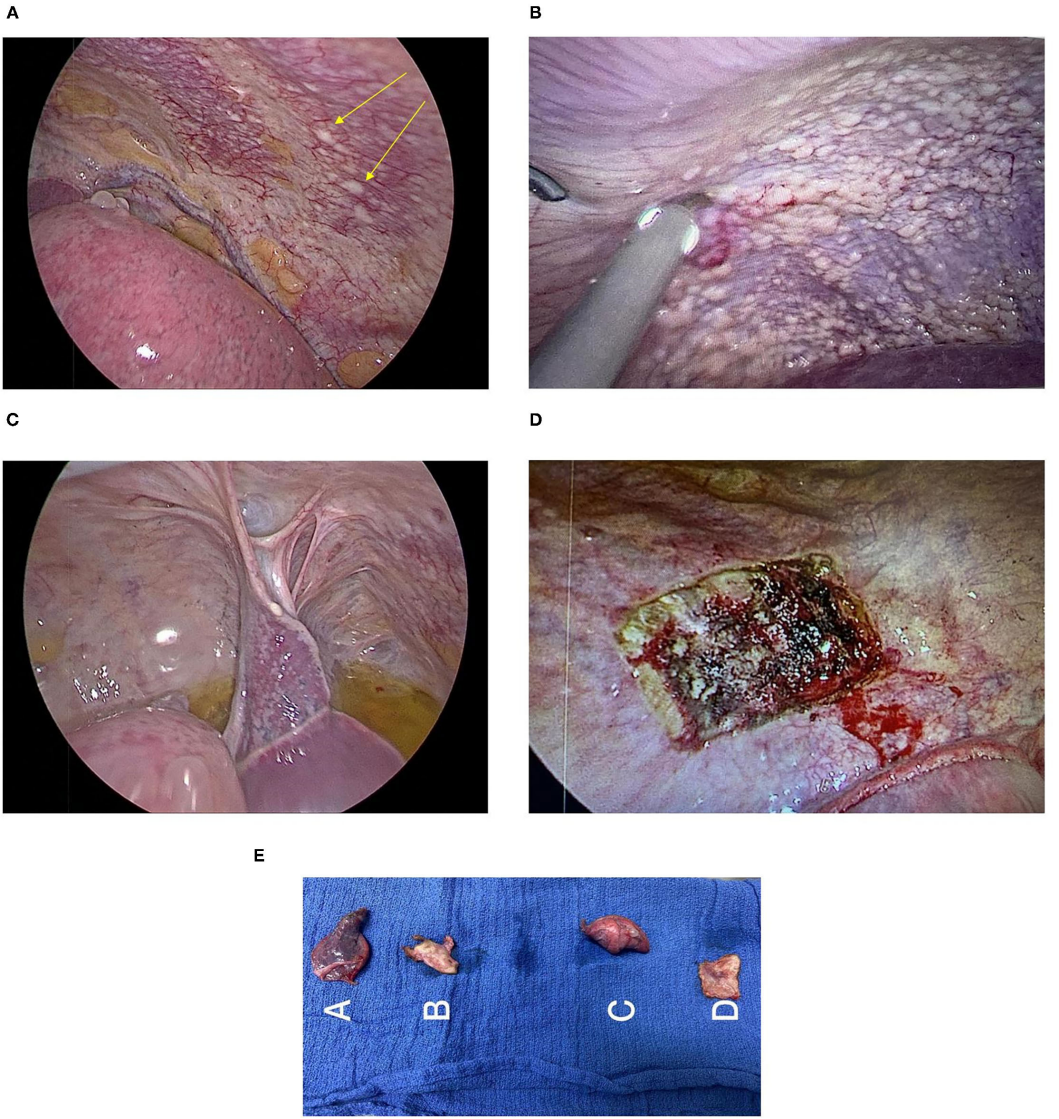
**(A)** Visceral and parietal pleura lesions. **(B)** Lesions on the peritoneal surface of the diaphragm. **(C)** Laparoscopic view of the peritoneal surface of the diaphragm shows whitish lesions with ascites. **(D)** Site of parietal pleura biopsy. **(E)** Biopsies: A-Lung and visceral pleural biopsy, B-Parietal Pleural Biopsy, C-Lung and visceral pleural biopsy, and D-Parietal Pleural Biopsy.

Subxiphoid uniportal VATS showed multiple disseminated scattered lesions on the left parietal and visceral pleura (Figure 4A). On laparascopy, multiple scattered lesions over the diaphragm (Figure 4B), liver and peritoneum were seen in addition to a small amount of ascites (Figure 4C) not detected before on Ultrasound or CT scan.

Wedge biopsy of the parietal pleura (Figure 4D) and other biopsies (Figure 4E) were obtained from lesions of the mentioned sites.

Pathology report after pleural and peritoneal biopsies was consistent with previous diagnosis of foci of atypical mesothelial proliferation favorable of malignant mesothelioma, epithelioid subtype with low grade features.

Peritoneal fluid cytology showed sheets of almost unremarkable mesothelial cells with few lymphocytes.

## Diagnostic Assessment

Malignant pleural mesothelioma was suspected to be the underlying pathology due to the unexplained chronic shortness of breath, recurrent pleural effusions and strong family history of similar presentation.

The patient underwent right-sided uniportal VATS for exploration and obtaining biopsies. Pathology reports showed atypical malignant mesothelial proliferation, epithelioid subtype with low-grade features and without infiltration to underlying tissue (lung or chest wall).

^18^F-FDG PET/CT whole body scan was performed for staging and evaluating contralateral lung and extrathoracic involvement. Unfortunately, the result was inconclusive: there was no evidence of hypermetabolic pulmonary or pleural nodules.

The PET/CT scan failed to evaluate for contralateral lung or extrathoracic metastasis, even to confirm the positive pathology biopsy results on the right side.

Consequently, a left pleural biopsies were taken through subxiphoid uniportal VATS, and a laparoscopy was done on the same session to assess for peritoneal mesothelioma.

Obtaining the diagnosis and determining the prognosis of Malignant Pleural Mesothelioma in a young patient with no clues of malignancy on CT scans was the real challenge in this case, the patient is stage IV because it involves the ipsilateral pleural surfaces, contralateral pleura and peritoneal surface, but still it doesn't involve the underlying lung or chest wall. That's why staging was confusing for this case and doesn't truly reveal the prognosis.

As for the therapeutic interventions–pharmacologic mainly-which were followed in the treatment of our patient: on 12/2020, a right sided PLEURX drain was inserted temporarily after taking pleural and peritoneal biopsies. Later, the patient was referred to an oncology center, at which she was given six cycles of chemotherapy (Alimta + Cisplatin), and the last cycle was on 7/2021. Each cycle was followed by folic acid and B12 supplementation.

The patient lost 5 kilograms of her weight during chemotherapy, and her hemoglobin dropped to 9g/dl, but soon after finishing the cycles, she had gained her weight, and her Hb level was 12 g/dl.

On April 17, 2021, a follow up Chest X-ray showed significant decrease in the amount of pleural effusion (Figure 1C). While on September, 2021, a follow up ^18^F-FDG PET/CT whole body scan showed: minimal right-sided pleural effusion with atelectatic and emphysematous lung changes with no significant metabolic activity, stable right pleural tiny calcified foci and mild-moderate left sided pleural effusion without metabolic activity, and this result confirmed patient improvement after chemotherapy. As for the follow-up plan, the next PET scan will be done in January, 2022.

## Timeline Table

**Table:** 

**Date**	**Procedure**
August/2020	Chest CT scan: huge right pleural effusion, left mediastinal shift, thickened pleura and collapsed right lung
October/2020	Chest CT scan: large right-sided pleural effusion and small left pleural effusion
October/2020	Uniportal VATS right pleural effusion evacuation, pleural and lung biopsies
November/2020	^18^F-FDG PET/CT scan was inconclusive: there was no evidence of hypermetabolic pulmonary or pleural nodules.
November/2020	Pathology report/pleural biopsies: malignant mesothelioma epithelioid subtype
December/2020	Follow up Chest CT scan revealed large right-sided pleural effusion, left-sided mediastinal shift and mild left pleural effusion, but no significant lymphadenopathy was noted. No masses or definite pleural thickening or nodularity.
December/2020	Subxiphoid Uniportal VATS left pleural, laparoscopic peritoneal biopsies and insertion of a temporary right PleurX
December/2020	Pathology report from pleural tissue was consistent with previous report and peritoneal fluid cytology revealed sheet of almost unremarkable mesothelial cells
January/2021-July/2021	Six cycles of chemotherapy (Alimta + Cisplatin) were given to the patient. Each cycle was followed by folic acid and B12 supplementation.
September/2021	Follow up ^18^F-FDG PET/CT scan showed minimal right-sided pleural effusion with atelectatic and emphysematous lung changes with no significant metabolic activity

## Discussion

### Familial Non-asbestos Related Mesothelioma

Malignant pleural mesothelioma is a truly rare disease with an incidence of 3,000 cases per year in the United States (7). The primary and the most important cause of pleural mesothelioma is asbestos exposure (8), which happens after a prolonged exposure to asbestos with an average age at diagnosis: 71–74 years (9), and this leads us to the following points that should be taken into consideration when encountering a patient with mesothelioma:

- Mesothelioma isn't always related to asbestos exposure, several familial cases with germ-line mutation in BAP1 tumor suppressor gene have been described to have increased susceptibility to malignant pleural mesothelioma and uveal melanoma (3). This is what was found in our patient, which wasn't related to asbestos exposure, instead, the patient had a heterozygous BAP1 mutation with family history of the disease and the mutation.
- Age of diagnosis in our patient was: 38, that is approximately 34 years before the average age of diagnosis as mentioned above, and this may provide us with a clue that familial malignant pleural mesothelioma presents in younger ages, with possible different behavior than the classical diffuse malignant pleural mesothelioma.
- Our patient had strong family history related to MPM and BAP1 mutation, her maternal and paternal uncles were highly suspected to have Malignant mesothelioma, with the second having a confirmed BAP1 mutation and malignancy disease. Both relatives suffered from recurrent effusions, were diagnosed at their 50 s, received no treatment except drainage until death.

### Diagnosis and Staging

^18^F-FDG PET/CT scan was considered as an optimal modality for MPM staging and evaluating extrathoracic metastasis (10). However, in the case of the high risk individuals such as patients with positive family history of BAP-1 mutations like in BAP-1 Tumor Predisposition Syndrome (BAP1-TPDS) VATS should be considered even in negative PET scan uptake as this patient was approached because false negative outcomes have been reported (11).

Regarding the explanation of the false-negative result of PET/CT scan, the low grade of the tumor, and the low metabolic activity of its cells is possibly attributed to be the underlying cause of no FDG uptake by the tumor cells. Although old age was also considered as an independent predictor, this is not the case in our young 38 years old patient (12).

Additionally, PET/CT scan role isn't only limited to pre-operative staging and extrathoracic disease evaluation, but also it has additional prognostic role based on the intensity of FDG-uptake. Our patient had no uptake, which may be considered to have better prognosis (13).

VATS is the gold standard for diagnosing MPM, providing and securing enough tissue biopsy for pathological examination, in addition to its therapeutic role in the effective drainage of pleural effusion through PleureX drain insertion (14, 15). This approach allows earlier diagnosis and more efficient management that hopefully will lead to better prognosis.

Familial BAP1 associated mesothelioma is considered to have better prognosis with higher survival rates, slow progression and non-aggressive tumor behavior. It more commonly occurs at earlier age which is thought to play a role in its' better prognosis and more commonly involves the peritoneal cavity compared to the classic sporadic diffuse malignant mesothelioma (16, 17).

According to IACLS staging system (18), our patient is stage IV with visceral pleura, contralateral lung and peritoneal involvement, who lived for 5 years with minimal vague symptoms prior to diagnosis and had a good response to the chemotherapy treatment.

The IACLS staging system for the diffuse malignant mesothelioma may not accurately reflect the prognosis of familial BAP1 MPM. As in our patient, the prognosis is incompatible with expected sporadic mesothelioma survival rate (5 year survival: 47%-familial vs. 6.7%-sporadic) (19).

All examined lesions' biopsies were low grade atypical mesothelial cells, not infiltrating the underlying tissues. This leads us to suspect that these nodular lesions are not secondary or metastatic, instead, they're all more likely to be multiple foci of primary malignant mesothelial proliferations.

The extent of the disease and the extrathoracic metastasis were explored by doing left sided (contralateral) thoracoscopy and laparoscopy, which showed atypical mesothelial proliferation and mesothelial cells in left pleural and peritoneal fluids, respectively. This diagnostic step conducted in this reported case confirms the superior value of subxiphoid Uniportal Video Assisted Thoracoscopy VATS value, biopsies examination and histopathology reports over PET scan to obtain a proper staging of MPM.

This leads us to the importance of good history taking from the patient, our patient has strong family history of BAP1 mutation and recurrent pleural effusions for 5 years, hence, the patient's physicians role wasn't limited to performing thoracentesis treatment without obtaining a diagnosis and recommended specialist consult and follow up.

Lastly, we would like to highlight the importance of establishing screening programs and genetic counseling for patients with family history of MPM. Significant efforts should be made in order to encourage families with known history of BAP1 mutation to undergo screening for MPM early detection and to face their fear of being stigmatized.

It's also crucial to create a modified staging system for the better evaluation of BAP1 associated mesothelioma cases that will more clearly reflect the prognosis of the familial cases, moreover treatment options targeting BAP1 may have promising outcomes.

## Patient Perspective

Despite the obvious familial pattern of the disease and malignancy history in multiple family members in this case, there are challenges must be highlighted that may refrain other family members from further investigations or screening workup as demonstrated in the maternal uncle of our patient, these include; fear of being stigmatized and social problems that may arise from inappropriate social interaction, which may result in an inadvertent discrimination against certain families leading to precluding early detection of the disease and worsens prognosis.

## Data Availability Statement

The original contributions presented in the study are included in the article/supplementary material, further inquiries can be directed to the corresponding author.

## Ethics Statement

Written informed consent was obtained from the individual(s) for the publication of any potentially identifiable images or data included in this article.

## Author Contributions

MI and KT contributed to conception, design of the study, wrote the first draft of the manuscript, and wrote sections of the manuscript. MI, KT, FA, and IB contributed to data collection, data analysis, and interpretation. All authors contributed to manuscript revision, read, and approved the submitted version.

## Conflict of Interest

The authors declare that the research was conducted in the absence of any commercial or financial relationships that could be construed as a potential conflict of interest.

## Publisher's Note

All claims expressed in this article are solely those of the authors and do not necessarily represent those of their affiliated organizations, or those of the publisher, the editors and the reviewers. Any product that may be evaluated in this article, or claim that may be made by its manufacturer, is not guaranteed or endorsed by the publisher.
